# Clinical decision tool for CRT-P vs. CRT-D implantation: Findings from PROSE-ICD

**DOI:** 10.1371/journal.pone.0175205

**Published:** 2017-04-07

**Authors:** Victor Nauffal, Yiyi Zhang, Tanyanan Tanawuttiwat, Elena Blasco-Colmenares, John Rickard, Joseph E. Marine, Barbara Butcher, Sanaz Norgard, Timm-Michael Dickfeld, Kenneth A. Ellenbogen, Eliseo Guallar, Gordon F. Tomaselli, Alan Cheng

**Affiliations:** 1 Department of Medicine, Division of Cardiology, Johns Hopkins Medical Institutes, Baltimore, Maryland, United States of America; 2 Welch Center for Prevention, Epidemiology and Clinical Research, Johns Hopkins Bloomberg School of Public Health, Baltimore, Maryland, United States of America; 3 Department of Medicine, University of Maryland, Baltimore, Maryland, United States of America; 4 Department of Medicine, Virginia Commonwealth University, Richmond, Virginia, United States of America; Cedars-Sinai Medical Center, UNITED STATES

## Abstract

**Background:**

Cardiac resynchronization therapy (CRT) devices reduce mortality through pacing-induced cardiac resynchronization and implantable cardioverter defibrillator (ICD) therapy for ventricular arrhythmias (VAs). Whether certain factors can predict if patients will benefit more from implantation of CRT pacemakers (CRT-P) or CRT defibrillators (CRT-D) remains unclear.

**Methods and results:**

We followed 305 primary prevention CRT-D recipients for the two primary outcomes of HF hospitalization and ICD therapy for VAs. Serum biomarkers, electrocardiographic and clinical variables were collected prior to implant. Multivariable analysis using Cox-proportional hazards model was used to fit the final models. Among 282 patients with follow-up outcome data, 75 (26.6%) were hospitalized for HF and 31 (11%) received appropriate ICD therapy. Independent predictors of HF hospitalization were atrial fibrillation (HR = 1.8 (1.1,2.9)), NYHA class III/IV (HR = 2.2 (1.3,3.6)), ejection fraction <20% (HR = 1.7 (1.1,2.7)), HS-IL6 >4.03pg/ml (HR = 1.7 (1.1,2.9)) and hemoglobin (<12g/dl) (HR = 2.2 (1.3,3.6)). Independent predictors of appropriate therapy included BUN >20mg/dL (HR = 3.0 (1.3,7.1)), HS-CRP >9.42mg/L (HR = 2.3 (1.1,4.7)), no beta blocker therapy (HR = 3.2 (1.4,7.1)) and hematocrit ≥38% (HR = 2.7 (1.03,7.0)). Patients with 0–1 risk factors for appropriate therapy (IR 1 per 100 person-years) and ≥3 risk factors for HF hospitalization (IR 23 per 100-person-years) were more likely to die prior to receiving an appropriate ICD therapy.

**Conclusions:**

Clinical and biomarker data can risk stratify CRT patients for HF progression and VAs. These findings may help characterize subgroups of patients that may benefit more from the use of CRT-P vs. CRT-D systems.

**Trial registration:**

ClinicalTrials.gov NCT00733590

## Introduction

Cardiac resynchronization therapy pacemakers (CRT-P) and CRT defibrillators (CRT-D) both impart improvements in mortality in groups of patients with dyssynchronous heart failure [1,2], but within the group, there is variability as to whether pump failure or ventricular arrhythmias (VAs) account for the greatest proportion of one’s overall mortality risk [3, 4]. Discerning a patient’s primary mode of death is important as this may guide the proper use of CRT systems and potentially avoid risks such as inappropriate ICD shocks that have been associated with poor outcomes as well as increased risk of infections with subsequent ICD pulse generator exchanges [5,6]. We assessed predictors of HF hospitalizations and ICD therapy for VAs in primary prevention CRT-D recipients within the Prospective Observational Study of Implantable Cardioverter Defibrillators (PROSE-ICD) to identify factors that may characterize subgroups of patients that may benefit more from the use of CRT-P vs. CRT-D systems.

## Methods

### Study design, population, and phenotyping

PROSE-ICD is a multi-center prospective observational study conducted at four U.S. institutions that enrolled 1,189 consecutive patients with heart failure and depressed ejection fraction eligible for implantation of primary prevention ICD or CRT-D devices from December 2003 to January 2013. The study design and protocol have been previously described [7]. Briefly, all patients underwent a comprehensive history and cardiovascular physical examination, electrocardiographic (ECG) evaluation, cardiac imaging, and blood draw at the time of device implantation. Patients were routinely followed at 6 months intervals and soon after any patient-perceived ICD therapy. Serum protein biomarkers were measured in baseline fasting samples and were selected based on pathophysiologic relevance in HF [8]. Four inflammatory biomarkers were tested using high sensitivity ELISA including high sensitivity C-reactive protein (HS-CRP), interleukin 6 (IL6), interleukin 10 (IL10), and soluble tumor necrosis factor alpha receptor IIa (TNF-αRIIa). These inflammatory biomarkers were chosen based on evidence in the literature for a mechanistic role in the pathogenesis of heart failure. CRP is an acute phase reactant that is produced by hepatocytes in response to rising levels of circulating Il-6 [8]. Elevated levels of CRP have been shown to be an independent predictor of arrhythmias [9] as well as associated with progressive myocardial dysfunction in patients with chronic heart failure [10]. Il-6 induces hypertrophy in cardiac myocytes and exerts its effects through propagating adverse remodeling. TNF-alpha additionally has been associated with progression of heart failure and LV dilation through activation of matrix metalloproteinases [11, 12]. Finally, IL-10 is an anti-inflammatory cytokine that keeps the pro-inflammatory milieu in check and may serve a protective role [13]. Whether certain biomarkers are more intimately related to arrhythmogenesis or pump failure in heart failure remains to be determined. Serum levels of N-terminal pro-brain natriuretic peptide (NT pro-BNP) as well as markers of myocardial injury including cardiac troponin T (cTnT), cardiac troponin I (cTnI) and creatine kinase MB (CK-MB) were also measured. This analysis leveraged the 305 participants with a CRT-D system. The Johns Hopkins Hospital, University of Maryland, Virginia Commonwealth University Hospital and Washington Hospital Center-Medstar Health Institutional Review Boards approved the study and all participants provided written informed consent before enrollment.

### Device implantation and programming

All patients undergoing CRT-D implantation met current practice guidelines at the time of implant [2]. Right ventricular leads were targeted to the RV apex and left ventricular pacing leads were targeted to a lateral or posterolateral branch of the great cardiac vein with special care to avoid apical placement of the LV lead given reports of inferior outcomes with LV apical pacing [14]. Device programming was not mandated by a specific protocol but rather left to the discretion of the implanting physician. Atrio-ventricular and ventriculoventricular delays were not routinely optimized but programmed to allow for fully biv-paced ventricular complexes [15, 16].

### Outcome assessment, adjudications and patient follow-up

The two primary endpoints were HF hospitalization and appropriate ICD therapy for VAs. HF hospitalization was defined as hospitalization with signs and symptoms of HF substantiated by a primary diagnosis of HF. Hospitalization data were evaluated at follow-up visits and verified by individual patient chart review as well as follow-up with patients’ corresponding primary physicians. At the time of regular follow-up visits or following patient-perceived ICD therapy, all stored electrograms were collected and adjudicated separately by two board-certified electrophysiologists. In case of disagreement, a third adjudicator provided a final decision. All events resulting in ICD therapy were adjudicated for the initial detected rhythm, rate, type and number of therapies delivered as well as resulting rhythm post-therapy. Appropriate therapy events were defined as ICD therapy (eg, antitachycardia pacing (ATP) or shocks) delivered for VAs with no prior ICD therapy for supraventricular tachycardia in the preceding 24 hours. Time-to-event for each of the primary endpoints was calculated as time from device implant to first occurrence of the corresponding endpoint. Patients were censored at five years follow-up or following consent withdrawal, device explant/inactivation, heart transplant or left ventricular assist device implant (LVAD) whichever came first. Twenty-three (7.5%) patients were lost-to-follow up after device implant and were excluded from the current analysis. The excluded participants were overall very similar to the study population with notable statistically significant differences including older age, worse kidney function and lower baseline hemoglobin (**Table A in S1 File**).

### Statistical analysis

Descriptive statistics for categorical variables are summarized as frequency (%). Continuous variables are presented as mean ± SD or median (interquartile range) for Gaussian and non-Gaussian distributions, respectively. Continuous variables were transformed into binary variables using pre-specified cut-offs either derived from clinical and laboratory recognized criteria or from cut-offs validated in the literature. With the exception of cardiac enzymes where normal reference cut-offs were used, cut-offs for biomarker data were assigned according to the 75^th^ percentile (**Table B in S1 File**). Univariable Cox proportional hazards models were used to assess the association of clinical, echocardiographic and ECG parameters as well as basic laboratory and serum biomarker data with the two primary endpoints. Variables with a p-value <0.1 in univariable analysis were included in the final models. Multivariable Cox proportional hazards model with a backward selection method was used to fit the final models. Only variables with a p<0.05 with the primary endpoints after backward regression were retained in the final models. The two final models generated were used separately to calculate the predicted five-year probability of HF hospitalization and appropriate therapy for individual subjects. Spearman rank correlation coefficients were calculated to assess correlation between the predicted five-year probabilities. A simple scoring system for each endpoint was developed by assigning a point score of 1 for each variable included in the final models. The final score was calculated by adding all score points. Kaplan-Meier methodology with the log-rank significance test was used to compare cumulative hazard rates of each endpoint across its respective score categories. Consecutive score categories with no significant difference were combined into one category. Finally, association of score categories for the two endpoints with all-cause mortality was assessed using Kaplan-Meier methodology with the log-rank significance test. The validity of the proportional hazards assumption was verified by using the log-negative-log(*Survival*[t]) plot. The Breslow method was used to handle event ties. Cox-Snell residuals were calculated and used to test goodness-of-fit of the final models (**Figures A and B in S1 File**). Discriminative power was assessed by the Harrell’s c-statistic. A two-sided p<0.05 was considered statistically significant. All analyses were done using STATA 12 (StataCorp LP; College Station, TX).

## Results

Among 305 primary prevention CRT-D recipients, 282 had complete follow-up data over a median of 5 (3.4–5) years. Mean age at enrollment was 62.1±12.3 years with males and African Americans constituting 62.8% and 26.2% of the study population, respectively (**Table 1**). Patients had predominantly NYHA Class II and III status with 92.9% of patients with an implanted atrial lead. Device programming included the use of anti-tachycardia pacing (ATP) in 55.7% of patients. The mean lowest cut-off rate for detection of VAs was 186.4±13.3bpm. There was no statistically significant differences in device programming characteristics between participants who received appropriate therapy during follow-up and those who did not **(Table C in S1 File)**.

**Table 1:** Baseline characteristics—All cohort.

Baseline Characteristics	All * [N = 282]
Age [years]	62.1 ± 12.3
Male [%]	177 [62.8]
African American [%]	74 [26.2]
BMI [kg/m^2^]	29.4 ± 6.2
Systolic blood pressure [mmHg]	121 [110–135.3]
Heart rate [beats/min]	76.0 ± 15.8
QRS [ms]	147.4 ± 26.6
LBBB [%]	147 [52.1]
NYHA Class [%]	
I	30 [10.6]
II	109 [38.7]
III	139 [49.3]
IV	4 [1.4]
Ejection fraction [%]	20.7 ± 7.4
NT pro-BNP [ng/L]	2,800 [1,800–4,300]
Ischemic Cardiomyopathy [%]	103 [36.5]
Diabetes [%]	104 [36.9]
Hypertension [%]	168 [59.6]
Dyslipidemia [%]	122 [43.3]
Atrial fibrillation [%]	80 [28.4]
Smoking [%]	190 [67.4]
Beta Blocker [%]	252 [89.4]
ACE-I [%]	197 [69.9]
ARB [%]	63 [22.3]
Loop Diuretic [%]	203 [72.0]
Aldosterone Antagonist [%]	85 [30.1]
Statin [%]	178 [63.1]
EGFR [ml/min/1.73m^2^]	71.8 ± 23.6
Sodium [meq/L]	139.1 ± 3.0
Hemoglobin [g/dl]	13.2 ± 1.8
Device Type [%]	
Dual Chamber BiV Pacemaker	262 [92.9]
Single Chamber BiV Pacemaker	20 [7.1]
Number of Zones Programmed [%]	
1	156 [55.3]
2	111 [39.4]
3	15 [5.3]
ATP Programmed [%]	157 [55.7]
Lowest Rate Cut-off [beats/min]	186.4 ± 13.3

* Only 282 patients with follow-up data post-implant are included.

ACE-I: angiotensin converting enzyme inhibitor; ARB: angiotensin receptor blocker; ATP: anti-tachycardia pacing; BMI: body mass index; EGFR: estimated glomerular filtration rate; LBBB: left bundle branch block; NYHA: New York heart association; NT pro-BNP: N terminal pro-brain natriuretic peptide.

**Fig 1:**
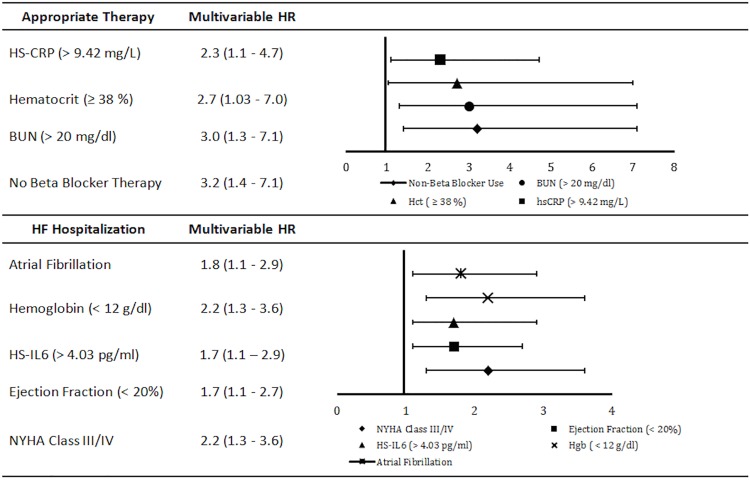
Multivariable hazard ratios for appropriate therapy and HF hospitalization.

**Fig 2:**
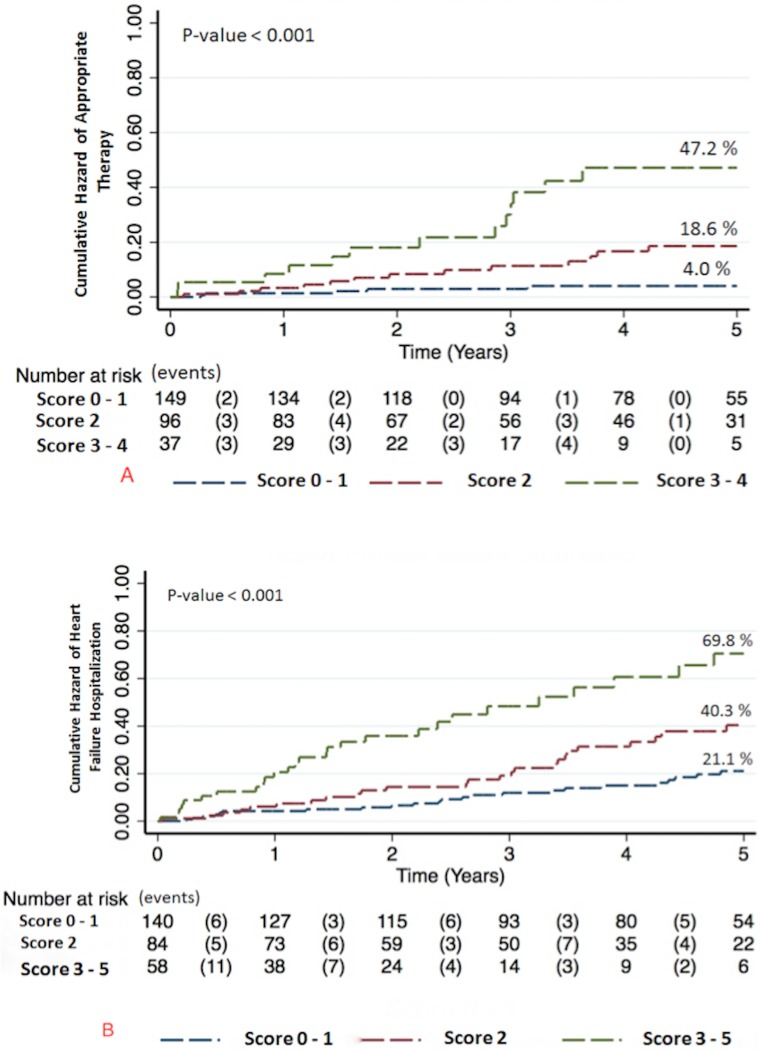
Cumulative hazard according to score categories. (A) Cumulative hazard of appropriate therapy according to score categories. (B) Cumulative hazard of HF hospitalization according to score categories.

Over the follow-up period, 31 patients received appropriate ICD therapy for VAs (20 shocks, 11 ATP). Ventricular tachycardia was the underlying rhythm in 87.1% cases with the remaining due to ventricular fibrillation. Median heart rate of treated arrhythmias was 215bpm (interquartile range, 194-240bpm). Median time to appropriate therapy was 1.7 years [0.8–3]. Male gender, no beta blocker therapy, elevated inflammatory markers (i.e., HS-CRP and HS-IL6), and elevated blood urea nitrogen (BUN) were associated with a significantly increased risk of appropriate ICD therapy (**Tables 2–4**). On the other hand, advanced NYHA class III/IV and anemia were associated with a non-significant trend for decreased risk of appropriate ICD therapy. In multivariable analysis, four variables were identified as significant predictors of appropriate ICD therapy: BUN >20mg/dL (HR = 3.0 (1.3,7.1); p = 0.01), HS-CRP >9.42mg/L (HR = 2.3 (1.1,4.7); p = 0.03), no beta blocker therapy (HR = 3.2 (1.4, 7.1); p = 0.006) and hematocrit ≥38% (HR = 2.7 (1.03,7.0); p = 0.04) (**Fig 1**). The Harrell’s c-statistic of the final model was 0.76 (0.67, 0.85). Three distinct score categories were identified: category 1 (score 0–1 (n = 149, 52.8%)), category 2 (score 2 (n = 96, 34.1%)) and category 3 (score 3–4 (n = 37, 13.1%)). Five-year cumulative risk of appropriate therapy was 4%, 14.6% and 47.2% for score categories 1, 2 and 3, respectively (p< 0.001) (**Fig 2A**).

**Table 2:** Unadjusted hazard ratios [HR] for appropriate therapy and HF hospitalization–clinical characteristics and medications.

Clinical Characteristics *	Unadjusted HR -Appropriate Therapy	Unadjusted HR—HF Hospitalization
Age, > 70 [years]	1.4 [0.7–3.0]	0.9 [0.5–1.5]
Male † ‡	2.6 [1.1–6.3]	0.6 [0.4–0.99]
African American	0.4 [0.2–1.2]	1.2 [0.7–1.9]
BMI, ≥ 30 [kg/m^2^]	1.6 [0.8–3.1]	1.3 [0.8–2.0]
Systolic blood pressure, < 110 [mmHg]	1.8 [0.9–3.8]	1.4 [0.9–2.4]
Heart rate, >75 [beats/min] ‡	0.8 [0.4–1.5]	1.6 [1.01–2.6]
QRS >150 [ms]	1.2 [0.6–2.4]	1.3 [0.8–2.1]
QTc >450 [ms]	1.6 [0.8–3.3]	1.4 [0.8–2.2]
LBBB	0.6 [0.3–1.2]	0.9 [0.6–1.4]
NYHA Class III/IV ‡	0.9 [0.4–1.8]	2.5 [1.6–4.1]
Ejection fraction, < 20 [%] ‡	1.3 [0.7–2.7]	1.8 [1.1–2.8]
Ischemic Cardiomyopathy	0.7 [0.3–1.4]	1.1 [0.7–1.7]
Diabetes	1.03 [0.5–2.2]	1.5 [0.9–2.4]
Hypertension	1.0 [0.5–2.0]	1.02 [0.7–1.6]
Dyslipidemia	1.5 [0.7–3.00	1.1 [0.7–1.8]
Atrial fibrillation	1.8 [0.9–3.8]	1.5 [0.9–2.4]
History of Myocardial Infarction	0.8 [0.3–1.8]	0.9 [0.5–1.6]
History of Percutaneous Coronary Intervention	0.7 [0.2–2.0]	1.4 [0.8–2.5]
History of CABG	0.6 [0.2–1.8]	1.5 [0.9–2.5]
Family History of Sudden Cardiac Death	0.7 [0.3–1.6]	1.2 [0.8–2.0]
Family History of Myocardial Infarction	0.7 [0.3–1.4]	1.3 [0.8–2.1]
Smoking	0.9 [0.4–1.9]	0.7 [0.4–1.1]
Device Type		
CRT-D, no atrial lead	Reference	Reference
CRT-D, with atrial lead	0.6 [0.2–2.1]	0.6 [0.3–1.2]
Number of Zones Programmed		
1	Reference	Reference
2	1.03 [0.5–2.2]	1.1 [0.7–1.8]
3	2.6 [0.9–7.8]	0.7 [0.2–2.2]
Lowest Rate Cut-off [bpm]	0.98 [0.96–1.0]	1.0 [0.99–1.02]
**Medications**		
Beta Blocker †	0.3 [0.1–0.6]	0.7 [0.4–1.5]
ACE-I	0.6 [0.3–1.3]	0.96 [0.6–1.6]
ARB	0.9 [0.4–2.2]	1.2 [0.7–2.1]
Calcium Channel Blocker	1.9 [0.7–5.0]	0.9 [0.4–2.02]
Digoxin	1.2 [0.6–2.6]	1.0 [0.6–1.7]
Thiazide	1.6 [0.6–4.2]	1.6 [0.9–3.0]
Loop Diuretic ‡	1.7 [0.7–4.2]	2.1 [1.2–3.8]
Aldosterone Antagonist	0.8 [0.3–1.8]	1.2 [0.7–2.0]
Statin	1.03 [0.5–2.2]	0.8 [0.5–1.3]
Aspirin	1.4 [0.7–3.0]	1.4 [0.9–2.3]
Clopidogrel	0.5 [0.1–2.0]	1.4 [0.8–2.6]

* Thresholds for continuous variables were derived from normal reference ranges or from cut-offs validated in the literature.

† P < 0.05 for Appropriate therapy.

‡ P < 0.05 for HF hospitalization.

ACE-I: angiotensin converting enzyme inhibitor; ARB: angiotensin receptor blocker; BMI: body mass index; CABG: coronary artery bypass graft; LBBB: left bundle branch block; NYHA: New York Heart Association.

**Table 3:** Unadjusted hazard ratios [HR] for appropriate therapy and HF hospitalization–serum biomarkers.

Serum Biomarkers *	Univariate HR Appropriate Therapy	Univariate HR HF Hospitalization
HS-CRP, >9.42mg/L † ‡	2.6 [1.2–5.3]	2.4 [1.5–3.8]
HS-IL6, >4.03pg/mL † ‡	2.5 [1.2–5.1]	2.4 [1.5–3.8]
TNF-αRIIa, >4863pg/mL ‡	1.5 [0.7–3.3]	1.7 [1.02–2.9]
IL10, >2.6pg/ml	0.96 [0.4–2.2]	1.1 [0.7–1.9]
NT pro-BNP, >4,300ng/L ‡	1.3 [0.6–3.0]	1.7 [1.1–2.8]
cTnT >28ng/L ‡	1.5 [0.7–3.2]	1.9 [1.2–3.0]
cTn I >34ng/L	1.4 [0.7–2.8]	1.1 [0.7–1.8]
CK-MB >2.5ng/mL	1.8 [0.8–3.7]	1.2 [0.7–1.8]

* Thresholds for biomarker data were assigned according to the 75^th^ percentile except in the case of cardiac injury markers were clinical and laboratory recognized cut-offs were used.

† p<0.05 for Appropriate therapy.

‡ p<0.05 for HF hospitalization.

HS-CRP: high sensitivity c-reactive protein; HS-IL6: high sensitivity interleukin 6; TNF-αRIIa: tumor necrosis factor alpha receptor 2a; IL10: interleukin 10; NT pro-BNP: N terminal pro-brain natriuretic peptide; cTnT: cardiac troponin T; cTn I:cardiac troponin I; CK-MB: creatine kinase MB.

**Table 4:** Unadjusted hazard ratios [HR] for appropriate therapy and HF hospitalization–laboratory data.

Laboratory Data *	Univariate HR Appropriate Therapy	Univariate HR HF Hospitalization
Sodium <135 mEq/L ‡	2.6 [0.9–7.5]	2.5 [1.2–4.9]
Potassium <4 mEq/L	0.8 [0.3–1.8]	1.02 [0.6–1.7]
Chloride <98 mEq/L	1.7 [0.6–4.9]	1.6 [0.8–3.2]
BUN >20 mg/dl †	3.7 [1.6–8.6]	1.3 [0.8–2.0]
Creatinine >1.2 mg/dL ‡	1.6 [0.7–3.3]	1.7 [1.1–2.8]
EGFR <45 ml/min/1.73m^2^ ‡	1.3 [0.4–3.6]	2.0 [1.1–3.7]
Calcium <9 mg/dL	1.3 [0.6–2.7]	1.2 [0.7–2.0]
Magnesium <1.5 mEq/L	1.04 [0.4–3.0]	1.2 [0.7–2.3]
WBC count < 4500 /μL	0.8 [0.2–3.3]	1.4 [0.7–3.0]
RBC count <3.8 x 10^6^ /μL ‡	0.7 [0.2–2.4]	1.9 [1.1–3.3]
Hemoglobin <12g/dL ‡	0.4 [0.1–1.3]	2.2 [1.3–3.5]
Hematocrit <38%	0.4 [0.2–1.1]	1.5 [0.95–2.4]
MCV >100 fL	2.2 [0.5–9.2]	1.4 [0.4–4.5]
RDW >14.5% ‡	1.9 [0.9–3.9]	1.8 [1.1–2.8]
Platelet count >200,000 /μL ‡	1.8 [0.9–3.7]	1.6 [1.03–2.6]

* Thresholds for continuous variables are defined according to clinical and laboratory defined criteria

† p<0.05 for Appropriate therapy.

‡ p<0.05 for HF hospitalization.

BUN: blood urea nitrogen; EGFR: estimated glomerular filtration rate; MCV: mean corpuscular volume; RBC: Red Blood Cells; RDW: red cell distribution width; WBC: white blood cells.

Seventy-five patients were hospitalized for HF. Median time to HF hospitalization was 2 years [0.8–3.4]. Female gender, elevated heart rate, advanced NYHA class III/IV, low ejection fraction and loop diuretic therapy were associated with an increased risk of HF hospitalization (**Table 2**). In addition, elevated inflammatory markers (e.g., HS-CRP, HS-IL6 and TNF-αRIIa), elevated NT-pro BNP, elevated cTnT, hyponatremia, anemia and renal dysfunction were more likely in patients hospitalized for HF (**Tables 3 and 4**). In multivariable analysis, five variables were identified as significant predictors of HF hospitalization: atrial fibrillation (HR = 1.8 (1.1,2.9); p = 0.030, NYHA class III/IV (HR = 2.2 (1.3,3.6); p = 0.002), ejection fraction <20% (HR = 1.7 (1.1,2.7); p = 0.03), HS-IL6 >4.03pg/ml (HR = 1.7 (1.1,2.9); p = 0.03) and hemoglobin <12g/dL (HR = 2.2 (1.3,3.6); p = 0.003) (**Fig 1**). The Harrell’s c-statistic of the final model was 0.7 (0.63, 0.77). Three distinct score categories were identified: category 1 (score 0–1 (n = 140, 49.6%)), category 2 (score 2 (n = 84, 29.8%)) and category 3 (score 3–5, (n = 58, 20.6%)). Five-year cumulative risk of HF hospitalization was 21.1%, 40.3% and 69.8% for score categories 1, 2 and 3, respectively (p<0.001) (**Fig 2B**).

**Fig 3:**
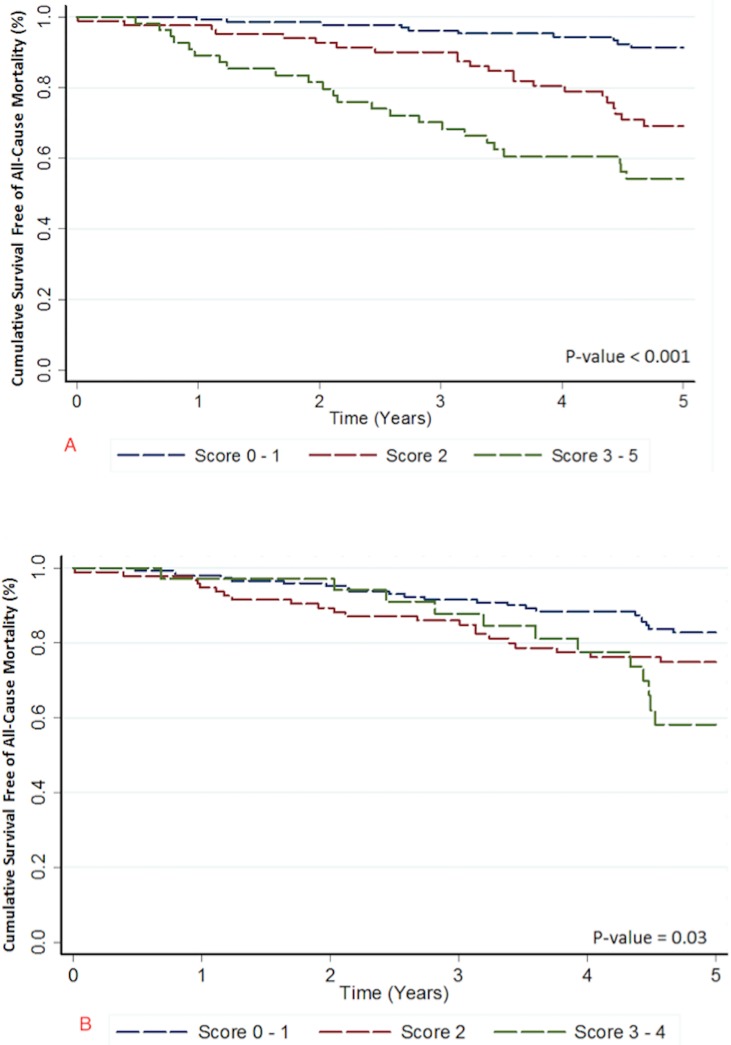
Survival free of all-cause mortality according to score categories. (A) Survival free of all-cause mortality according to HF hospitalization score category (B) Survival free of all-cause mortality according to appropriate therapy score category.

All-cause mortality was strongly associated with HF score category with greater risk for death with higher HF scores (**Fig 3A**). The relationship between death and appropriate therapy score categories, on the other hand, was not as distinct (**Fig 3B**). More specifically, when comparing the appropriate therapy score categories 2 and 3, there was no significant difference in all-cause mortality (p = 0.13) but each HF score category imparted a different risk for all-cause mortality.

**Fig 4:**
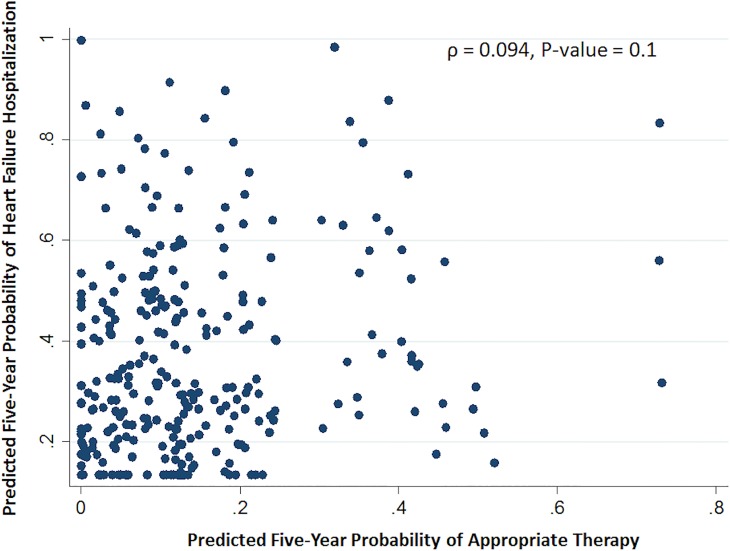
Scatter plot of five-year probability of HF hospitalization versus appropriate therapy.

**Fig 5:**
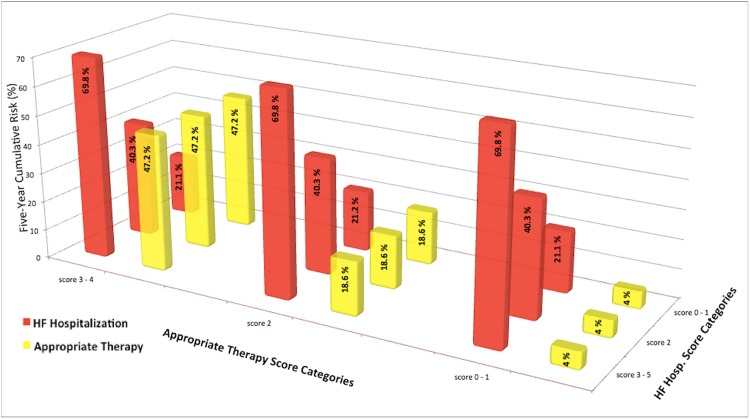
Five-year cumulative risk of HF hospitalization and appropriate therapy stratified by score categories.

No correlation between the predicted five-year probability for each of the two primary endpoints based on the derived models was found (Spearman ρ = 0.094, p = 0.1) (**Fig 4**). Furthermore, 23 participants fell simultaneously in score category 1 for appropriate therapy and score category 3 for HF hospitalization and represent a sub-group of patients at very low-risk of arrhythmic events (Incidence Rate (IR) 1 per 100 person-years) and high-risk of HF progression (IR 23 per 100 person-years) (**Fig 5**).

## Discussion

CRT has proven to be highly effective in the management of dyssynchronous heart failure both through its delivery of resynchronizing pacing as well as its treatment of life-threatening VAs. While groups of patients eligible for this therapy are at risk for both HF hospitalization and VAs, individuals within the group have varying degrees of risk for each of these events. Our study aimed to better identify factors that could discriminate between these two endpoints. Utilizing an extensively phenotyped group of primary prevention CRT-D recipients, we found the variables increasing the risk for HF hospitalization were distinct from those associated with an increased risk of VAs. By synthesizing these observations into risk score model constructs, we were able to identify a group of patients with high risk for HF hospitalization and low risk for VAs. In fact, 23 (8.2%) participants were concurrently in score category 1 for appropriate therapy and score category 3 for HF hospitalization. They represent a sub-group of patients at very low risk of arrhythmic events (IR 1 per 100 person-years) and high risk of HF progression (IR 23 per 100 person-years) that may potentially not derive incremental survival benefit from ICD therapy.

### Predictors of HF hospitalization

Predictors of HF hospitalization included IL6 levels >4.03pg/ml, NYHA class III/IV, EF <20%, hemoglobin <12g/dL and atrial fibrillation and were consistent with findings from other CRT cohorts. Patients with atrial fibrillation were excluded from most CRT trials with the exception of the RAFT trial, which in a post-hoc analysis failed to show benefit from CRT in this particular sub-group [17]. This has been further corroborated by several other observational studies [18, 19]. Additionally, a dose-response effect has been described relating the burden of atrial fibrillation to adverse outcomes in CRT patients [20]. Anemia, a frequently comorbid condition in HF (26% of CHARM study participants), has been consistently identified as a poor prognostic marker [21]. Whether anemia is a marker of poor baseline function or a direct contributor to HF morbidity and mortality has not yet been clearly elucidated [22].

Circulating cytokines are elevated in HF and contribute to the overall pro-inflammatory state. IL6 has been shown to be an independent predictor of HF morbidity and mortality [23, 24] as well as implicated in the development of anemia of chronic disease in HF patients [25].

### Predictors of appropriate ICD therapy

Blood urea nitrogen levels >20mg/dL, no beta blocker therapy, hematocrit ≥38% and HS-CRP >9.42mg/L predicted appropriate ICD therapy in our cohort. In an analysis of the COMPANION trial, renal dysfunction and absence of beta blocker therapy were independent predictors of sudden cardiac death and appropriate ICD shocks, respectively [26]. A role for inflammatory cytokines including HS-CRP in the prediction of ventricular arrhythmia risk has been previously described in the literature [27]. We have previously shown in a sub-population of our cohort who underwent contrast enhanced cardiac magnetic resonance imaging that the presence of heterogeneous myocardial tissue (gray zone) and elevated HS-CRP are the only two independent predictors of appropriate ICD shock [9]. As mentioned earlier, anemia is a common comorbid condition with HF that portends adverse survival. The increased risk of VAs seen in patients with hematocrit ≥38% might reflect a better functional status of these patients thus prolonging their time at risk for VAs.

### Prior studies comparing CRT-P and CRT-D

In light of the lack of robust clinical trial data comparing CRT-P and CRT-D, the majority of the evidence on the comparative effectiveness of CRT-P and CRT-D is derived from retrospective analysis of CRT registry data [28, 29]. Notable differences in baseline characteristics between CRT-P and CRT-D recipients have been shown. CRT-P recipients are more likely to be elderly, female, have non-ischemic HF, renal dysfunction, anemia, poorer functional class, wider QRS duration and no history of prior VAs when compared to their CRT-D counterparts. Hence, it is not surprising that reported outcomes of patients with CRT-P vs. CRT-D in these studies are conflicting. In patients with Class IA indication for CRT implantation, Morani et al. found CRT-D to be an independent predictor of long-term survival [28] while Looi et al. reported a non-significant trend for increased survival at one year with defibrillator therapy in CRT patients that attenuated with longer follow-up. Furthermore, in non-responders no survival benefit was witnessed at any of the follow-up times [29].

The COMPANION trial was the only randomized clinical trial that enrolled NYHA III/IV HF patients to CRT-P or CRT-D and was only powered to compare each of these arms to medical therapy but not to each other [30]. However, rates of the different pre-specified endpoints have been reported for each of the CRT arms despite no accompanying formal comparative statistical testing. In both the CRT-P and CRT-D arms, the reported cumulative incidence of the primary endpoint of all-cause mortality or any hospitalization at 12 months was 56%. Furthermore, when analyzing the secondary endpoint of death from or hospitalization for cardiovascular causes CRT-P decreased the risk by 25% when compared to best medical therapy while CRT-D decreased the risk by 28% with the two therapy arms having completely overlapping Kaplan Meier curves.

### Limitations

There are several limitations affecting this analysis. First, this was a relatively small cohort of CRT-D individuals studied and further studies comparing direct outcomes of patients with CRT-D and CRT-P devices will be informative. However, the findings from our analysis align with prior studies of larger groups of patients. Nevertheless, validation of these findings will be necessary in larger populations. Second, there was no standardization of CRT-D device programming as the majority of patients were enrolled prior to the MADIT-RIT study. With that said, the median rate of VAs resulting in defibrillator therapy was 215bpm (interquartile range, 194–240) which reflects a predominance of ICD therapy for fast life-threatening VAs. In addition, there was no significant difference in programming strategies between patients who received appropriate ICD therapy and those who did not. Third, serum biomarkers were only measured at baseline and may not accurately reflect the levels around the time of ICD therapy or HF hospitalization. The study design mandated that patients at the time of enrollment did not have an acute illness in the hope of having baseline inflammatory markers reflective of chronic levels. Fourth, optimal guideline directed medical therapy for heart failure at maximal tolerated doses was encouraged in our study but data on drug dosing was unfortunately not collected. Finally, our relatively small sample size did not allow us to perform a stratified analysis by the underlying cause of cardiomyopathy.

## Conclusion

HF is a complex syndrome orchestrated by multiple complex and overlapping pathways that can result in modes of death secondary to pump failure or VAs. Refining the risk of either of these endpoints in patients with dyssynchronous heart failure would improve guidance on the type of CRT system that stands to result in greatest patient benefit. By applying baseline clinical and biomarker parameters into a series of risk scores, an individual’s risk for HF progression and VAs can be quantified in a way that can possibly improve decision-making. Further validation of our findings in larger cohorts is needed to ensure generalizability and facilitate adoption of our models in clinical practice.

## Supporting information

S1 File Supplemental Material. (DOCX)
