# Supplementary material for: Clinical decision tool for CRT-P vs. CRT-D implantation: Findings from PROSE-ICD
Source: PLoS One. 2017 Apr 7;12(4):e0175205. doi: 10.1371/journal.pone.0175205 (PMC5384669; doi:10.1371/journal.pone.0175205)
Supplement: S1 File — (DOCX) [file pone.0175205.s001.docx]

**SUPPLEMENTAL MATERIAL**

**Supplemental Methods**

All biomarker measurements were made in serum. The inflammatory biomarkers HS-CRP (ALPCO Diagnostics, Salem NH), IL-6 (R&D Systems, Minneapolis MN) and IL-10 (R&D Systems) were measured using high sensitivity ELISAs according to the manufacturer’s instructions. The lower limits of detection (LLOD) for CRP, IL-6, and IL-10 were 0.5 ng/ml, 0.036 pg/ml, and <0.5 pg/ml, respectively, with inter-assay coefficients of variation (CV) of 2.2%, 5%, and 6.3%, respectively. Soluble TNF-αRII is more stable than TNF-α and was measured to index levels of the cytokine using an ELISA (R&D Systems) with a LLOD of 0.6 pg/ml and inter-assay CV of 5.9%. NTPro-BNP was measured using an ELISA (ALPCO Diagnostics) with a LLOD of 5 fmol/ml and an inter-assay CV of 5.25%. Markers of myocardial injury CK-MB, myoglobin, cTnI and cTnT were measured using antibody-based electrochemiluminescence detection and patterned array sandwich ELISA (Meso Scale Discovery, Rockville MD). A multiplex platform was used to measure CK-MB, myoglobin and cTnI whereas a monoplex format was used in the case of cTnT with LLODs of 0.021 ng/ml, 0.075 ng/ml, 1.24ng/ml and 0.72 pg/ml, respectively. The intra- and inter-assay CVs for the cardiac injury platforms varied from 2.4 - 8.4% (mean 5.6%) and 4.3 – 9.5% (mean 7.8%), respectively.

| **Supplemental Tables**  **Table A – Baseline Characteristics of Participants with Complete Follow-up and Incomplete Follow-up**   \| **Baseline Characteristics** \| **Complete Follow-Up**  **[N = 282]** \|  \| **Incomplete Follow-Up**  **[N = 23]** \| \| --- \| --- \| --- \| --- \| \| Age [years] † \| 62.1 ± 12.3 \|  \| 70.4 ± 12.4 \| \| Male [%] \| 177 [62.8] \|  \| 16 [69.6] \| \| African American [%] \| 74 [26.2] \|  \| 6 [26.1] \| \| BMI [kg/m^2^] \| 29.4 ± 6.2 \|  \| 30.5 ± 7.4 \| \| Systolic blood pressure [mmHg] \| 121 [110 - 135.3] \|  \| 120 [108 - 138] \| \| Heart rate [beats/min] \| 76.0 ± 15.8 \|  \| 80.5 ± 22.2 \| \| QRS [ms] \| 147.4 ± 26.6 \|  \| 150.3 ± 24.2 \| \| LBBB [%] \| 147 [52.1] \|  \| 12 [52.1] \| \| NYHA Class [%] \|  \|  \|  \| \| I \| 30 [10.6] \|  \| 2 [8.7] \| \| II \| 109 [38.7] \|  \| 7 [30.4] \| \| III \| 139 [49.3] \|  \| 14 [60.9] \| \| IV \| 4 [1.4] \|  \| 0 [0] \| \| Ejection fraction [%] \| 20.7 ± 7.4 \|  \| 19.8 ± 7.2 \| \| NT pro-BNP [ng/L] \| 2,800 [1,800 – 4,300] \|  \| 2,900 [2,060 – 4,900] \| \| Ischemic Cardiomyopathy [%] \| 103 [36.5] \|  \| 8 [34.8] \| \| Diabetes [%] \| 104 [36.9] \|  \| 7 [30.4] \| \| Hypertension [%] \| 168 [59.6] \|  \| 14 [60.9] \| \| Dyslipidemia [%] \| 122 [43.3] \|  \| 13 [56.5] \| \| Atrial fibrillation [%] \| 80 [28.4] \|  \| 9 [39.1] \| \| Smoking [%] \| 190 [67.4] \|  \| 15 [65.2] \| \| Beta Blocker [%] \| 252 [89.4] \|  \| 20 [87] \| \| ACE-I [%] \| 197 [69.9] \|  \| 12 [52.2] \| \| ARB [%] \| 63 [22.3] \|  \| 6 [26.1] \| \| Loop Diuretic [%] \| 203 [72.0] \|  \| 20 [87] \| \| Aldosterone Antagonist [%] \| 85 [30.1] \|  \| 4 [17.4] \| \| Statin [%] \| 178 [63.1] \|  \| 14 [60.9] \| \| EGFR [ml/min/1.73m^2^] † \| 71.8 ± 23.6 \|  \| 50.7 ± 20.1 \| \| Sodium [meq/L] \| 139.1 ± 3.0 \|  \| 139.3 ± 4.2 \| \| Hemoglobin [g/dl] † \| 13.2 ± 1.8 \|  \| 12.3 ± 2.2 \| \| Device Type [%] \|  \|  \|  \| \| Dual Chamber BiV Pacemaker \| 262 [92.9] \|  \| 21 [91.3] \| \| Single Chamber BiV Pacemaker \| 20 [7.1] \|  \| 2 [8.7] \| \| Number of Zones Programmed [%] \|  \|  \|  \| \| 1 \| 156 [55.3] \|  \| 9 [39.1] \| \| 2 \| 111 [39.4] \|  \| 14 [60.9] \| \| 3 \| 15 [5.3] \|  \| 0 [0] \| \| Lowest Rate Cut-off [beats/min] \| 186.4 ± 13.3 \|  \| 184.9 ± 11.7 \|   † P-Value<0.05 | |
| --- | --- | --- | --- | --- | --- | --- | --- | --- | --- | --- | --- | --- | --- | --- | --- | --- | --- | --- | --- | --- | --- | --- | --- | --- | --- | --- | --- | --- | --- | --- | --- | --- | --- | --- | --- | --- | --- | --- | --- | --- | --- | --- | --- | --- | --- | --- | --- | --- | --- | --- | --- | --- | --- | --- | --- | --- | --- | --- | --- | --- | --- | --- | --- | --- | --- | --- | --- | --- | --- | --- | --- | --- | --- | --- | --- | --- | --- | --- | --- | --- | --- | --- | --- | --- | --- | --- | --- | --- | --- | --- | --- | --- | --- | --- | --- | --- | --- | --- | --- | --- | --- | --- | --- | --- | --- | --- | --- | --- | --- | --- | --- | --- | --- | --- | --- | --- | --- | --- | --- | --- | --- | --- | --- | --- | --- | --- | --- | --- | --- | --- | --- | --- | --- | --- | --- | --- | --- | --- | --- | --- | --- | --- | --- | --- | --- | --- | --- | --- | --- | --- | --- | --- | --- | --- | --- | --- | --- |
| **Table B – Threshold Values for Continuous Variables** | |
| **Continuous Variables** | **Cut-off Values** |
| Age (years) | 70 |
| Body Mass Index (kg/m^2^) | 30 |
| Systolic Blood Pressure (mmHg) | 110 |
| Diastolic Blood Pressure (mmHg) | 70 |
| Heart Rate (beats/min) | 75 |
| QRS (ms) | 150 |
| QT_c_ (ms) | 450 |
| Ejection Fraction (%) | 20 |
| Sodium (meq/L) | 135 |
| Potassium (meq/L) | 4 |
| Chloride (meq/L) | 98 |
| Magnesium (mg/dL) | 1.5 |
| Calcium (mg/dL) | 9 |
| Estimated Glomerular Filtration Rate (ml/min/1.73m^2^) | 45 |
| Creatinine (mg/dL) | 1.2 |
| Blood urea nitrogen (mg/dL) | 20 |
| White blood cell count (/µL) | 4500 |
| Red blood cell count (x 10^6^/ μL) | 3.8 |
| Hemoglobin (g/dL) | 12 |
| Hematocrit (%) | 38 |
| Red cell distribution width (%) | 14.5 |
| Mean corpuscular volume (fL) | 100 |
| Platelets (x10^3^/µL) | 200 |
| Cardiac troponin T (ng/L) | 28 |
| Cardiac troponin I (ng/L) | 34 |
| Creatine kinase MB (ng/mL) | 2.5 |
| NT-pro-BNP (ng/L) | 4,300 |
| HS-CRP (mg/L) | 9.42 |
| HS-IL-6 (pg/mL) | 4.03 |
| Il-10 (pg/mL) | 2.6 |
| sTNF-αRIIa (pg/ml) | 4,863 |

| **Table C – Device Programming Characteristics of Participants With and Without Appropriate Therapy** | | | |
| --- | --- | --- | --- |
| **Device Programming Characteristics** | **Appropriate Therapy**  **during Follow-Up**  **[N = 31]** |  | **No Appropriate Therapy**  **during Follow-up**  **[N = 251]** |
| Number of Zones Programmed |  |  |  |
| 1 | 15 (48.4) |  | 141 (56.2) |
| 2 | 12 (38.7) |  | 99 (39.4) |
| 3 | 4 (12.9) |  | 11 (4.4) |
| Lowest Cut-off Rate (beats/min) | 182.6 ± 13.1 |  | 186.9 ± 13.3 |
| VF Zone Cut-off Rate | 201.3 ± 19.2 |  | 202.8 ± 17.4 |
| \| First VT Zone Cut-off Rate \| 184.4 ± 10.3 \|  \| 183/8 ± 11.4 \| \| --- \| --- \| --- \| --- \| | 184.4 ± 10.3 |  | 183.8 ± 11.4 |
| Second VT Zone Cut-off Rate | 158.8 ± 11.8 |  | 156.8 ± 13 |
| Device Type |  |  |  |
| Dual Chamber | 28 (90.3) |  | 234 (93.2) |
| Single Chamber | 3 (9.7) |  | 17 (6.8) |

**Supplemental Figures and Figure Legends**

**Figure Legends**

**Figure A. Nelson Aalen cumulative hazard versus Cox-Snell residual plot for testing goodness of fit for appropriate therapy prediction model.**

**Figure B. Nelson Aalen cumulative hazard versus Cox-Snell residual plot for testing goodness of fit for heart failure hospitalization prediction model.**


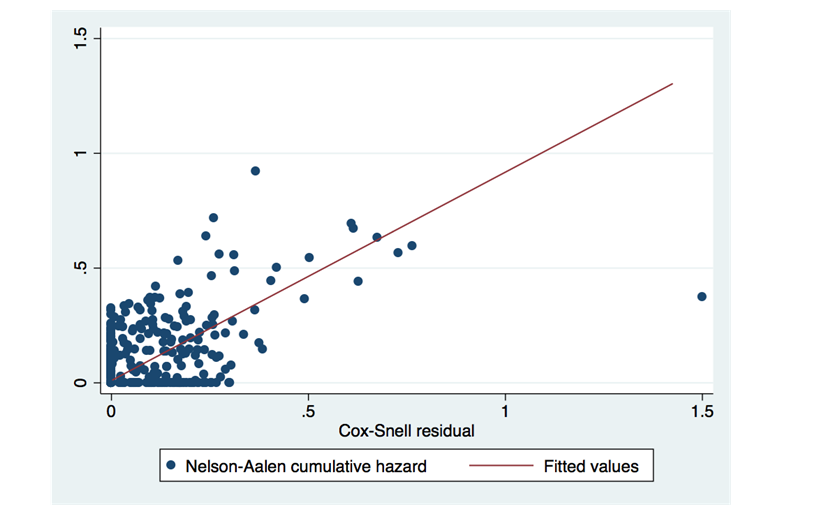


Figure 1


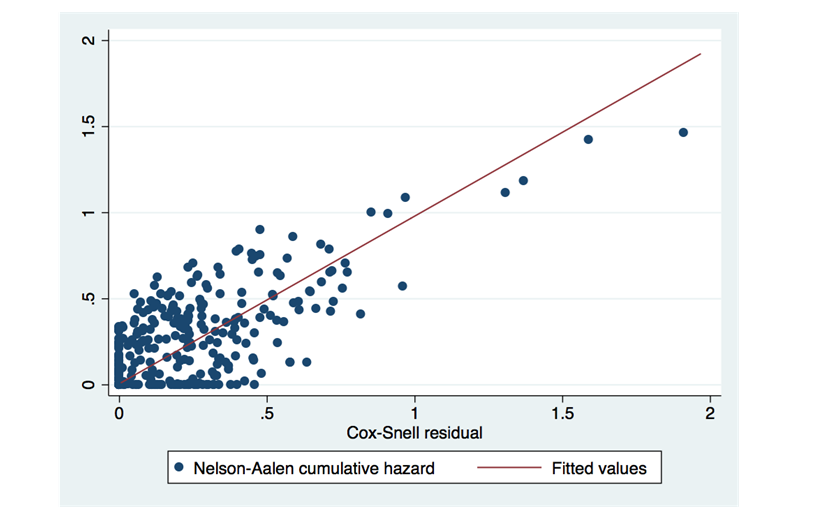


Figure 2
